# Investigation into Layer Number Effect on Breakdown Strength of Multi-Layer Polymer Films

**DOI:** 10.3390/polym14091653

**Published:** 2022-04-20

**Authors:** Liang Zhao, Binxiong Yu, Wei Shang

**Affiliations:** 1 Science and Technology on High Power Microwave Laboratory, Northwest Institute of Nuclear Technology, Xi’an 710024, China; yubinxiong@nint.ac.cn (B.Y.); shangwei@nint.ac.cn (W.S.); 2 College of Advanced Multidisciplinary Studies, National University of Defense Technology, Changsha 410073, China; 3 Key Laboratory of Physical Electronics and Devices of Ministry of Education, Xi’an Jiaotong University, No. 28 West Xianning Rd., Xi’an 710049, China

**Keywords:** layer number effect, solid insulation dielectrics, breakdown strength, multi-layer films

## Abstract

The layer number effect on electric breakdown strength (*E_BD_*) of multi-layer polymer films is investigated using 10-μm polypropylene (PP) films under a dc condition. The layer number, *n*, of the films during the test is as large as 120. It is observed that the relation between *E_BD_* and *n* conforms to a minus power law, i.e., *E_BD_*(*n*) = *E*_1′_*n*^−*a*^, where the power exponent, *a*, is 0.27, *E*_1′_ is a constant. By reviewing the experimental data in references, it is found that the power law holds true for different types of polymers in different test conditions, but the value of *a* varies from 0.072 to 0.5. The variation of *a* is explained in perspective of the discontinuous structures within films and those between films. A small value of *a* means a good purity level of the film, which is due to the decrease of the size of the inter-layer defects. A large value of *a* means a poor purity level of the films, which is due to the increase of the amount of intra-layer defects. Both factors influence the value of *a*, leading to the variation of *a*.

## 1. Introduction

Films are widely used in high-voltage (HV) devices and pulsed power systems due to their excellent insulation performance. For example, PZT/PZO-based multilayer films are used as energy-storage materials [1,2,3], polymer films rolled together with foils are used in capacitors [4], polymer films wound in big cylindrical electrodes are used as pulse forming line [5,6], polymer films wound in slim metal wires are used as high energy-density cables [7,8], polymer films between two foils stuck in HV devices are used as capacitive voltage dividers [9,10,11], and polymer films immersed in liquid are used as composite insulation materials in linear transformer devices (LTD) [12,13].

Recently, researchers have conducted plenty of investigations to explore the breakdown characteristics of films. For example, they paid attention to the temperature effect on the electric breakdown strength (*E_BD_*) of films [14,15,16], the thickness effect on *E_BD_* [17,18,19,20], the area effect on *E_BD_* [21,22,23,24], and the volume effect on *E_BD_* [25,26]. In addition, the thickness effect, the area effect, and the volume effect on *E_BD_* were summarized as a unified formula:

(1) $$E_{BD}\left(\zeta \right)=E_{1}\zeta^{-1/\beta }.$$

where *ζ* can represent either thickness, area, or volume; *E*_1_ is a constant; *β* is the shape parameter of the two-parameter Weibull distribution and is averaged to be 8 [27]. For thin films, the thickness effect on *E_BD_* is especially paid attention to since it is directly related to practical application, and the following formula is suggested:

(2) $$E_{BD}\left(d\right)=E_{1}d^{-a}.$$

where *a* = 1/*β*. A small *a* means that a film has a good purity level, as disclosed in [20,26].

Both Equations (1) and (2) present guidelines for practical insulation design. However, they are derived either from bulk polymers or from single-layer films. Whether Equation (2) holds true for multi-layer polymer films is the first question. If it does, is the power exponent a constant or not? This is the second question. If it is not a constant, what factors affect the power exponent? This is the third question. In this paper, these three questions are focused on. Following this section, Section 2 is devoted to the experimental research on the *E_BD_*-*n* relation with *n* as large as 120 under a dc test condition. Section 3 is devoted to a short review on the *E_BD_*-*n* relation in references. In Section 4, the mechanism responsible for the *E_BD_*-*n* relation is analyzed. The last section is for the conclusions of this paper.

## 2. Experimental Research on Relation between *E_BD_* and *n*

### 2.1. Experimental Setup

**Figure 1:**
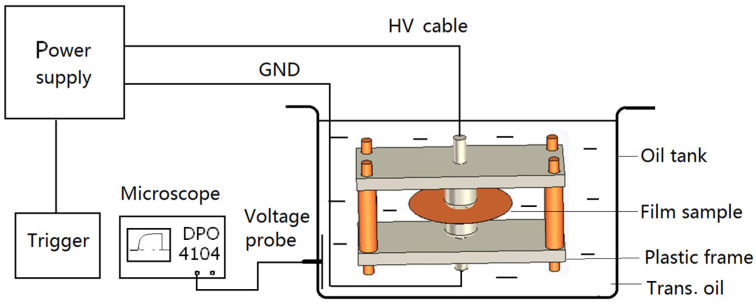
Experimental setup to test the *U_BD_* of *n*-layer films.

The experimental setup is based on a DC power supply, which can output a DC voltage up to 300 kV in a continuous way. The test sample is cut into a round shape with a diameter of 60 mm and placed between two stainless steel electrodes with a diameter of 20 mm, which are fixed by a plastic frame via polymer screws and the distance between the electrodes can be adjusted conveniently. The HV electrode is connected to the HV cable of the power supply; the low voltage electrode is connected to the ground (GND) of the power supply. The two electrodes, the frame, and the sample are placed in an oil tank filled with clean transformer oil to overcome edge discharges. In the DC power supply, there is a resistor about 2 MΩ to absorb the short current once breakdown takes place. The experimental setup is shown in Figure 1.

The samples are made of polypropylene (PP) films with a thickness of 10 μm. The brand is PHD BOPP. Table 1 lists the key parameters of this type of PP films. Before the test, the electrodes are polished with sandpapers of 1200 grit and cleaned with alcohol to refresh the electrode surface; then, *n*-layer films are placed together and loaded between the electrodes, immersed into transformer oil, and tested by a gradually increased voltage. Once breakdown takes place, the frame is taken out of the oil, the breakdown sample is replaced, and the next *n*-layer sample is tested. When five effective *U_BD_*-*n* data are acquired, the test for the *n*-layer films is finished. Then, the test for more-layer films is started by repeating the above procedures. 

### 2.2. Experimental Results

**Figure 2:**
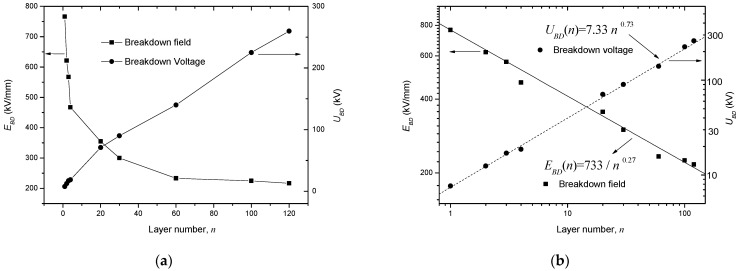
Experimental results of *U_BD_* and *E_BD_* dependent on *d*. (**a**) Raw experimental data; (**b**) fitted results in a log-log coordinate system.

Figure 2a shows the experimental results of the breakdown voltage (*U_BD_*) and the *E_BD_* dependencies on *d*, where *E_BD_* = *U*_BD_/(*nd*_0_) and *d*_0_ = 10 μm. From this figure, it is seen that the *U*_BD_ seems to increase linearly as *n* increases but *E_BD_* decreases sharply as *n* increases. 

It is assumed that the lay number effect on *E_BD_* of multi-layer films is a special case of the thickness effect on *E_BD_*, which still conforms to a minus power law. The deduction process is as follows. The total thickness of *n*-layer films is:

(3) $$d=nd_{0}.$$

By neglecting the interface factors and by inserting Equation (3) into Equation (2), one can obtain that:

(4) $$E_{BD}\left(n\right)=E_{1}^{\prime }n^{-a},$$

where *E*_1_^′^ = *E*_1_*d*_0_^−*a*^. 

In addition,

(5) $$U_{BD}\left(n\right)=E_{BD}\left(n\right)\cdot d=U_{1}n^{1-a},$$

where *U*_1_ = *E*_1_*d*_0_^1−*a*^. Equation (5) means that *U_BD_* and *n* also conform to a power relation. Based on Equations (4) and (5), the data in Figure 2a are re-plotted in a log-log coordinate and fitted linearly, as shown Figure 2b, which shows good agreement. 

## 3. Review of Different Groups of *E_BD_*-*n* Data

In order to present the most appropriate expression to describe the *E_BD_*-*n* dependency, different groups of *E_BD_*-*n* data in references are reviewed [12,13,24,29,30].

### 3.1. In Perspective of Layer Number

**Figure 3:**
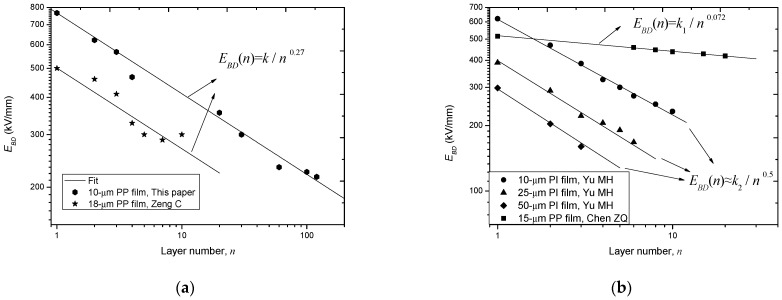
Re-plot and fit different groups of *E_BD_*-*n* data in log-log coordinate systems. (**a**) *E_BD_*-*n* data with moderate tendency; (**b**) *E_BD_*-*n* data with sharp and slow tendencies.

By reviewing the relevant references [12,13,24,29,30], we find that five groups of data are related to the layer number effect on *E_BD_*, which are plotted in Figure 3. From Figure 3, it is found that all these groups of data meet the minus power relation, but the power exponent is not a constant.

Table 2 lists the value of the power exponent, *a*, as well as the test information of these five groups of data, together with the data in this paper. From this table, it is seen that *a* ranges from 0.072 to 0.56 for different polymer films under different test conditions.

**Figure 4:**
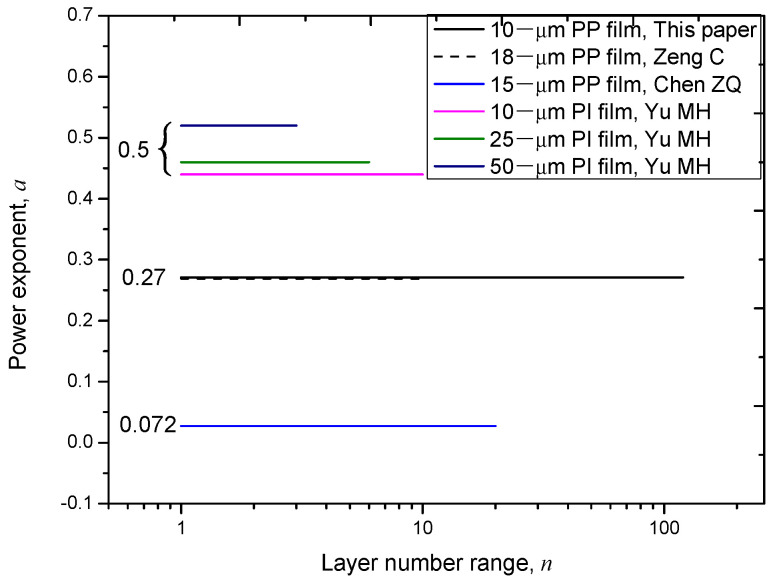
The value of power exponent, *a*, in a wide layer number range.

Figure 4 shows the value of *a* in a wide layer number range. From this figure, it is seen that the minus power relation of these six groups of data can be divided into three types according to the value of *a*:

(1) moderate dependency, which corresponds to a power exponent of 0.27;
(2) fast dependency, which corresponds to a power exponent about 0.5;
(3) slow dependency, which corresponds to a power exponent of 0.072.

### 3.2. In Perspective of Thickness

**Figure 5:**
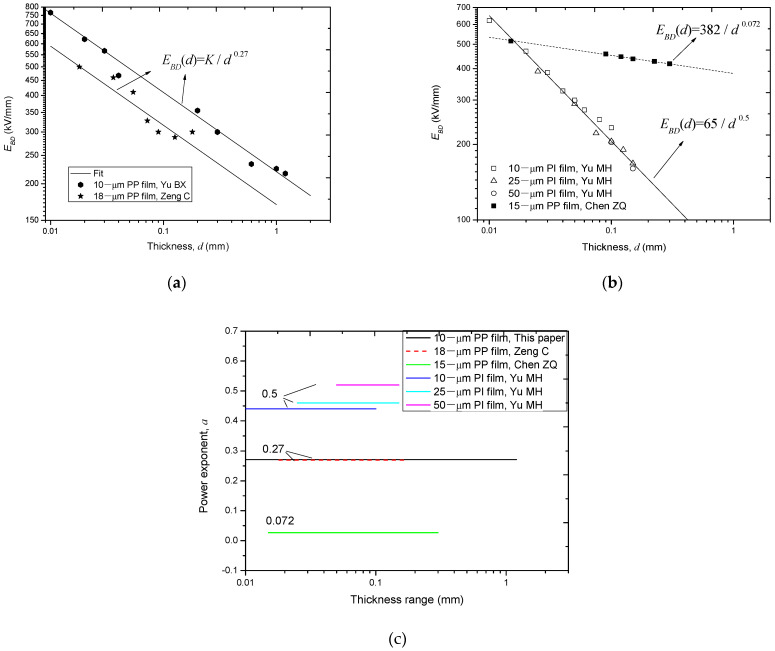
Re-plot and fit different groups of *E_BD_*-*d* data in log-log coordinate systems. (**a**) Moderate tendency of *E_BD_*-*d* data; (**b**) fast and slow tendency of *E_BD_*-*d* data; (**c**) comparison of the power exponent of the thickness effect in a wide thickness range.

As aforementioned in Section 2, the layer number effect on *E_BD_* of multi-layer films is assumed as a special case of the thickness effect on *E_BD_*. Thus, in order to further compare these groups of data, they are re-plotted and fitted using thickness as the argument, as shown in Figure 5. From Figure 5a, it is seen that the two moderate groups of *E_BD_*-*d* data get closer; from Figure 5b, it is seen that the three fast groups of *E_BD_*-*d* data overlap as one. In addition, the different value of *a* in a wide thickness range is plotted, as shown in Figure 5c. From Figure 5c, it is seen that the value of 0.27 validates in a much wider thickness range, i.e., 0.01–1 mm. All the three figures in Figure 5 give a support for the assumption that the lay number effect on *E_BD_* is a special case of the thickness on *E_BD_*.

As a sub-conclusion of this section, the relation between *E_BD_* and *n* conforms to a minus power law; but the power exponent is not a constant, which ranges from 0.07 to 0.5 and is averaged to be about 0.27.

## 4. Mechanism of the Layer Number Effect on *E_BD_*

Now, there is the last question: what factors affect the value of *a*? To answer this question, the breakdown mechanism of solid dielectrics is necessary to be reviewed. 

### 4.1. Review of Solid Dielectric Breakdown Mechanism

Solid dielectric breakdown can grossly be divided into electronic breakdown and thermal breakdown, electrical treeing breakdown, electro mechanical breakdown, and electrochemical breakdown [20]. The electronic breakdown is due to electron instability, which means that the gained energy for an electron transferring in a dielectric is larger than the lost energy, whereas the thermal breakdown is due to heat instability, which means that the heat-generating speed ratio is greater than the heat-dissipating speed ratio in a dielectric. When a dielectric is too thin, for example, a film, heat can hardly accumulate, thus, the mechanism of film breakdown can be classified into the electronic breakdown [28,29]. The electronic breakdown must meet the electron impact and ionization criterion, i.e.:

(6) $$\Delta I=qE_{op}\lambda ,$$

where Δ*I* is the ionization energy or the energy band gap; *q* is the electron charge (1.6 × 10^−19^ C); *E_op_* is the applied field; *λ* is the electron mean free path. For an ideal condition, the dielectrics are pure, Δ*I* is large and *λ* is small. Thus, only when *E_op_* increases beyond a large intrinsic breakdown field, *E__intrinsic_*, the breakdown can start. However, in practice, the dielectric is impure, containing a lot of defects such as impurities and voids. The existence of impurities will lead to the decrease of Δ*I*; the existence of voids will lead to the increase of *λ*. Both factors can lead to the decrease of *E_BD_*, i.e., the breakdown becomes easy. In addition, the more amount of the defects in a dielectric, the easier the breakdown becomes. In the perspective of the thickness effect on *E_BD_*, the more amount of the defects in unit thickness, the faster *E_BD_* decreases as *d* increases and the larger is the value of *a*. 

Simply, the less impure is a dielectric, the larger is the value of *a* in *E_BD_*(*d*) = *E*_1_*d*^−*a*^; instead, the more pure is a dielectric, the smaller is the value of *a*. This is the basic starting point to discuss the layer number effect on *E_BD_* of multi-layer films.

### 4.2. Factors Leading to Decrease of a

**Figure 6:**
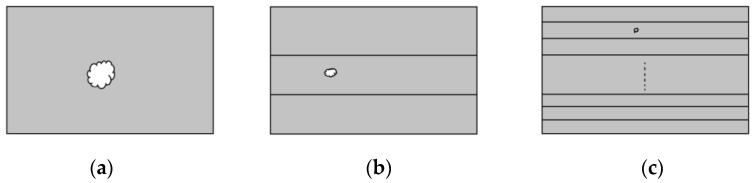
Schematics of void in solid dielectric with equal thickness. (**a**) Bulk material; (**b**) material composed by thick films; (**c**) material composed by thin films.

**Figure 7:**
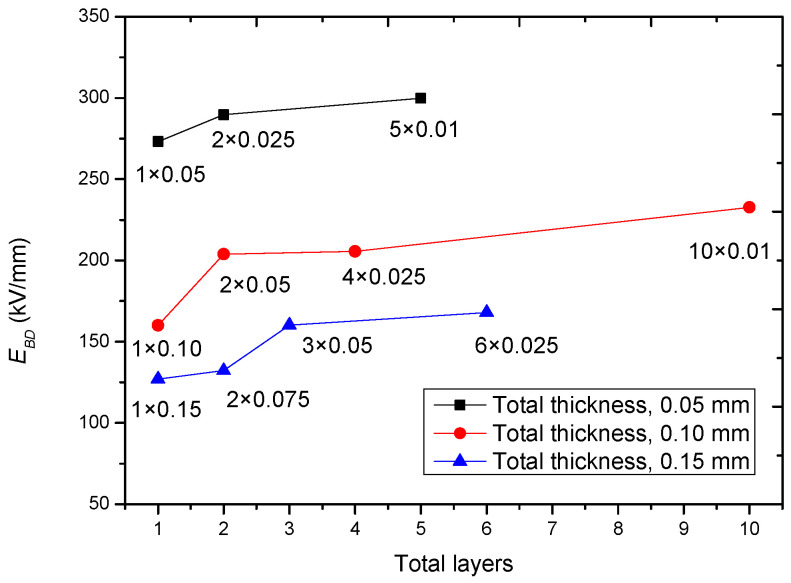
*E_BD_* of multi-layer films with a fixed total thickness dependent on different layers. The raw data are from [29].

Assume that there are three types of solid insulation materials with equal thickness: (a) bulk material; (b) material composed by thick films; (c) material composed by thin films, which is shown in Figure 6, and assume that all the three types of insulation materials have voids in them. Now, one can easily think out that as the thickness of single-layer film decreases, the size of the void becomes small, i.e., *λ* becomes small as the layer number of the multi-layer insulation material increases. Thus, the breakdown becomes difficult due to the increase of *E_BD_*, which can be verified by Figure 7. This can simply be considered as the increase of the pure level of the multi-layer insulation material. Now, once the total thickness of a multi-layer insulation material increases, the *E_BD_*-*n* or *E_BD_*-*d* dependency would become slow and would demonstrate a small power exponent of *a*.

**Figure 8:**
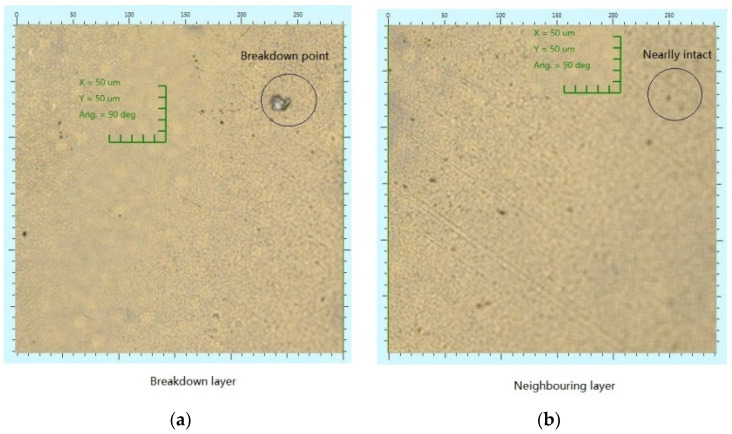
Microscopic images of the breakdown layer (**a**) and the neighboring layer (**b**) in a multi-layer film.

Aside from the advantage of purity, the multi-layer insulation material also tends to dissipate the discharge product, i.e., energy, plasma, heat, and high pressure, once one layer of film is breakdown. Thus, the breakdown channel cannot easily penetrate to the neighboring layer of film [30]. This is totally different from that of the bulk material, where the discharge product can easily accumulate in the vicinity of the tree tip, which is helpful to increase the breakdown channel once an electrical tree incepts [31,32]. Figure 8 shows the images of two neighboring films suffering a test. From these figures, it is seen that the first film has an obvious discharge point, whereas the second is basically intact. These two figures support such advantage of the multi-layer films.

### 4.3. Factors Leading to Increase of a

**Figure 9:**
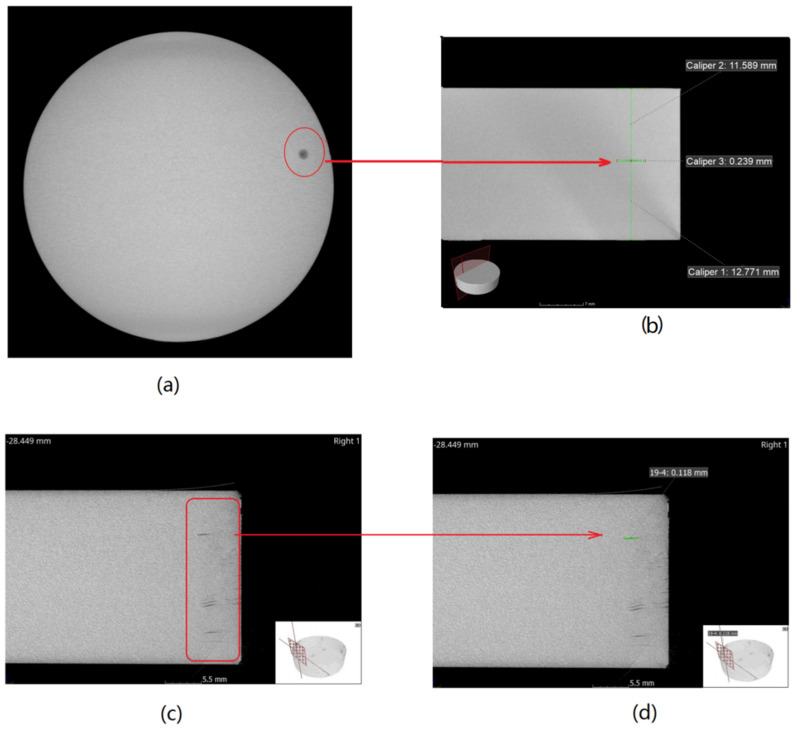
Cathode ray images of a disc multi-layer PP insulator, the total thickness is 20 mm; each layer has a thickness of 25 μm; the layers are stuck via PI film of 12.5 μm. (**a**) Top view of Sample A; (**b**) sectional view of Sample A with caliper; (**c**) sectional view of Sample B; (**d**) sectional view of Sample B with caliper.

However, the multi-layer insulation material has disadvantages that discontinuous structures can easily be introduced into the interface of different layers. Namely, it can produce more impurities and more voids, regardless of whether the films are united by sticking or by thermally shrinking. As aforementioned, the existence of defects can decrease the purity level of the multi-layer insulation material. In the perspective of thickness effect on *E_BD_*, the tendency of *E_BD_* on *d* would become fast, which corresponds to a large value of *a*. Figure 9 shows the computed tomography (CT) images of an insulator stuck by 1000-layer films with a thickness of 20 mm. From the figure, it is clearly seen that a lot of gas layers are formed between the film layers. The voids can easily decrease the purity of dielectric and accordingly increase the power exponent, *a*, for the thickness effect on *E_BD_*. 

As a sub-conclusion of this section, the power exponent, *a*, in the layer number effect on *E_BD_* of the multi-layer films is affected by two factors: the inter-layer defects and the intra-layer defects. As the thickness of single-layer decreases and the layer number increase, the size of inter-layer defects decreases; the purity level of the multi-layer polymer films increases; therefore, the value of *a* decreases. However, simultaneously, the amount of intra-layer defects increases due to the increase of film interfaces; therefore, the purity level of the multi-layer polymer films decreases and the value of *a* increases accordingly. Both factors play the role, which lead to variation of *a* in a wide range. 

## 5. Conclusions

There are two conclusions of this paper:

(1) The relation between *E_BD_* and *n* for the layer number effect on *E_BD_* conforms to a minus power law. The power exponent, *a*, range from 0.07 to 0.5 and is averaged to be 0.27.
(2) The value of *a* is affected by two factors: the inter-layer defects and the intra-layer defects. As the thickness of single-layer decreases, the inter-layer defects decrease in size and *a* decreases, whereas the intra-layer defects increases in amount and *a* increases. Both factors together lead to variation of *a* in a wide range.

## Figures and Tables

**Table 1:** Key parameters of the PP films [28].

Index	Value	Unit	Condition
Relative dielectric constant	2.2	/	23 °C: 50 Hz
	2.2		23 °C: 1 kHz
Dielectric loss factor	2 × 10^−4^	/	23 °C: 50 Hz
	2 × 10^−4^		23 °C: 1 kHz
Surface resistance	10^14^	Ω	23 °C
Breakdown field	700 400	V/μm	dcac, 23 °C, 50 Hz
Longitude tensile strength	175	MPa	Speed: 100%/min. 23 °C, 50% r.h
Horizontal tensile strength	290	MPa	
Longitude elastic modulus	2900	MPa	Speed: 100%/min. 23 °C, 50% r.h
Horizontal elastic modulus	4900	MPa	
Longitude elongation at break	165	%	Speed: 100%/min. 23 °C, 50% r.h
Horizontal elongation at break	55	%	
Longitude thermal shrinkage	2.5	%	120 °C in air, 15 min
Horizontal thermal shrinkage	0.6	%	
Density	0.91	g/cm^3^	23 °C
Water absorption	<−0.1	%	23 °C immersed in water for 4 days

**Table 2:** Different groups of *E_BD_*~*n* data.

Layer Number Range	Thickness per Layer/μm	Value of *a*	Test Object	Test Condition	Researcher /Year
1–10	18	0.27	PP films	Immersed in Glycerin; ns second pulse;	Zeng C/2014 [12,13,29]
1–10	10	0.44	PI films *	Immersed in transformer oil; ns second pulse;	Yu MH/2021 [30]
1–6	25	0.46			
1–3	50	0.56			
1–20	15	0.072	PP films	Immersed in transformer oil; dc voltage;	Chen ZQ/2022 [24]
1–120	10	0.27	PP films	Immersed in transformer oil; dc voltage;	This paper/2022

* PI: polyimide.

## Data Availability

Data presented in this study are available on request from the first author.
